# Neuroprotective potential of vitamin D_3_ against glucocorticoid-induced neurotoxicity: Focus on calcium-binding proteins and neuroinflammation in a rat model

**DOI:** 10.1016/j.ibneur.2026.09.016

**Published:** 2026-09-26

**Authors:** Ihor Shymanskyi, Olha Lisakovska, Anna Khomenko, Vasyl Bilous, Mykola Veliky

**Affiliations:** a Department of Biochemistry of Vitamins and Coenzymes, Palladin Institute of Biochemistry, National Academy of Sciences of Ukraine, Kyiv, Ukraine; b Department of Enzyme Chemistry and Biochemistry, Palladin Institute of Biochemistry, National Academy of Sciences of Ukraine, Kyiv, Ukraine

**Keywords:** Glucocorticoids, Vitamin D_3_, Neuroinflammation, Calcium homeostasis, Blood-brain barrier, Astrogliosis

## Abstract

Prolonged glucocorticoid (GC) therapy is frequently complicated by secondary vitamin D_3_ deficiency, which acts as a potential pathogenetic modulator of GC-induced neurotoxicity. This study investigated the role of disturbed calcium homeostasis and neuroinflammation in GC-induced brain damage and evaluated the protective potential of vitamin D_3_ (VD_3_, cholecalciferol). Female Wistar rats (n = 54) were divided into control, GC-treated (prednisolone, 5 mg/kg/day, 30 days, per os), and VD_3_-correction (1000 IU/kg/day, concurrent with GC) groups. Analysis of calcium-binding protein levels (calbindin-D28K, S100B, osteocalcin) and neurospecific markers (NF-L, MBP, GFAP) was performed using ELISA, Western blot, and confocal microscopy. Prolonged prednisolone administration caused significant systemic and local 25(OH)D_3_ deficiency, accompanied by a twofold reduction in osteocalcin protein content and elevated calbindin-D28K levels. Reactive astrogliosis, characterized by increased GFAP^+^ cell counts and S100B content, occurred alongside decline in NG2^+^ oligodendrocyte precursors. Significant neuroinflammation was recorded: TNF-α levels rose 1.62-fold in the cortex and 1.65-fold in the hippocampus, while IL-1β increased 2.66-fold and 2.40-fold, respectively. Furthermore, markedly increased serum levels of NF-L, S100B, GFAP, and MBP indicated alterations consistent with blood-brain barrier dysfunction and neuro-glial disintegration. VD_3_ therapy effectively restored serum markers (osteocalcin, S100B, MBP), suppressed astrocyte reactivity, and normalized cortical cytokine levels while partially replenishing NG2^+^ cell pool. Although its impact on hippocampal inflammation was limited, these findings demonstrate that maintaining adequate VD_3_ levels is paramount for preserving CNS integrity by stabilizing calcium buffering and suppressing neuroinflammation. Our results emphasize clinical importance of monitoring VD_3_ status to mitigate risk of glucocorticoid-induced neurotoxicity and associated neurological complications.

## Highlights

• Long-term prednisolone induces systemic and local vitamin D_3_ deficiency.
• Glucocorticoids disrupt Ca^2+^-binding proteins and trigger neuroinflammation.
• Vitamin D_3_ reduces cortical cytokines but shows limited hippocampal effect.
• Serum NF-L and MBP levels serve as sensitive biomarkers of BBB disruption.
• Vitamin D_3_ status is key for preserving CNS integrity during steroid therapy.

## Graphical Abstract

**Figure:**
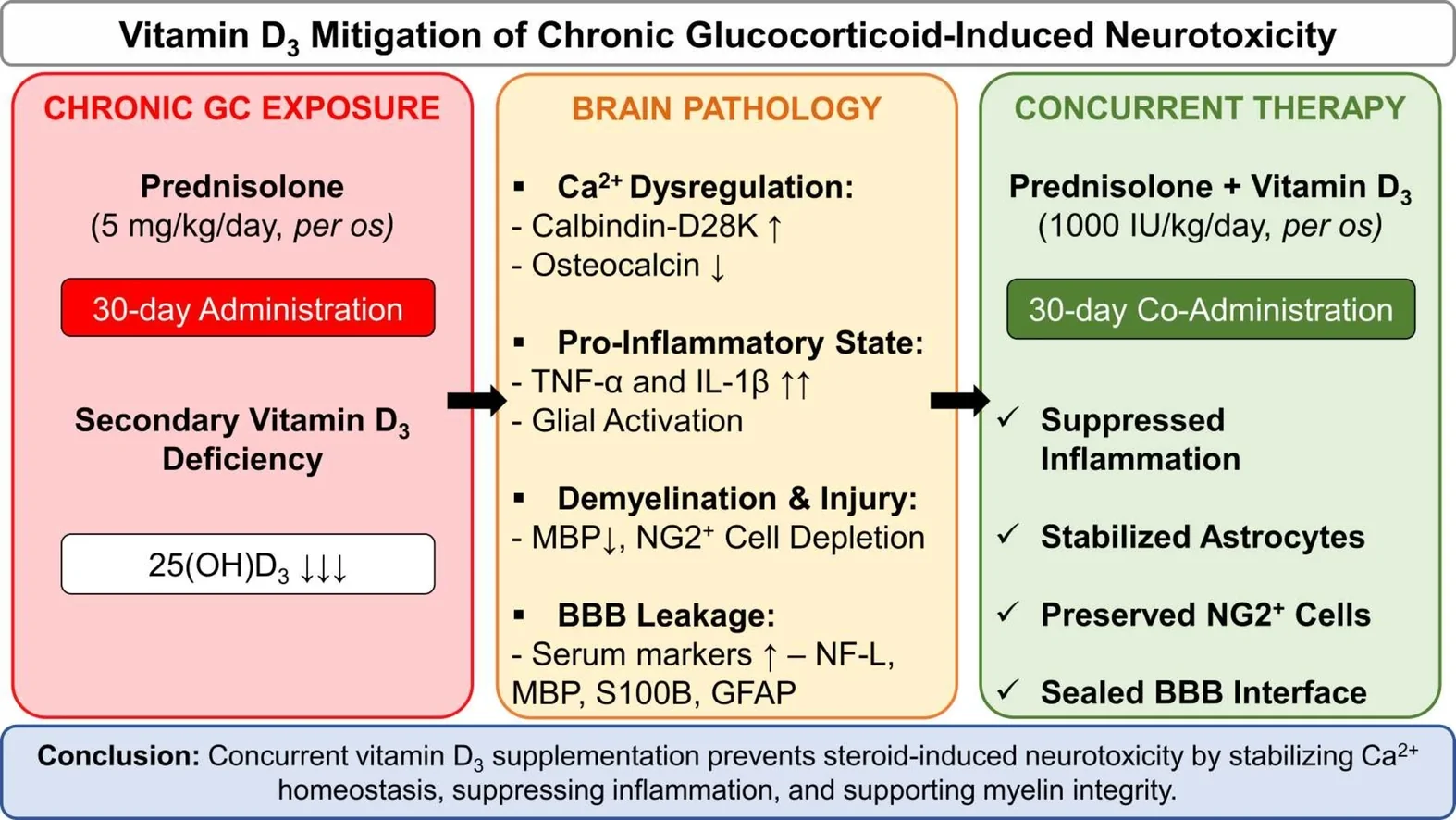

## 1 Introduction

Glucocorticoids (GCs) represent a class of steroid hormones synthesized in the adrenal cortex that regulate metabolism, predominantly carbohydrate homeostasis (Martinez et al., 2024). Due to their potent anti-inflammatory, immunosuppressive, and anti-shock properties, GCs remain cornerstone therapies for autoimmune and systemic conditions. These include rheumatoid arthritis, systemic connective tissue diseases, and severe allergic reactions, as well as chronic inflammatory pathologies affecting the gastrointestinal, cardiovascular, renal, and nervous systems (Martinez et al., 2024, Cummins and Goldstein, 2025, Reichardt et al., 2021). Given the multifaceted nature of their pharmacological effects, GCs interfere with the functioning of numerous organs and systems, influencing multiple metabolic pathways, which frequently precipitate significant side effects, including neurotoxicity (Shorey et al., 2023). This condition manifests as impairments in motor and sensory functions, emotional status, and integrative brain functions such as memory and learning, alongside potential psychiatric disturbances including insomnia, agitation, euphoria, and in some instances, psychosis or depressive behavior (Shorey et al., 2023, Koning et al., 2024).

A substantial factor exacerbating prolonged exposure to synthetic GCs is the development of a secondary vitamin D-deficient state, which acts as a crucial pathogenetic modulator in the progression of neuropathological alterations within the brain (Lisakovska et al., 2023). Vitamin D_3_ ( cholecalciferol), functioning as a neurosteroid, physiologically counteracts GC-associated neurotoxicity via specific receptor (VDR) signaling pathways (Korewo-Labelle et al., 2023, Su et al., 2025). Unlike ergocalciferol (vitamin D_2_), this specific form exhibits superior biological activity within brain tissues; however, vitamin D_3_ deficiency compromises this protective barrier (Menéndez and Manucha, 2024). This vulnerability renders neural tissue susceptible to the destructive influence of steroids, initiating a cascade of profound metabolic and functional disorganization within the central nervous system (CNS).

Current literature indicates that GC-related neurotoxicity exhibits distinct regional specificity, exerting maximal impact on the hippocampus, a structure characterized by the highest density of glucocorticoid receptors (Shorey et al., 2023). Specifically, sustained stress-induced glucocorticoid elevation triggers dendritic arborization atrophy and a reduction in pyramidal neuron volume, which correlate with observable cognitive impairments (Alfonso et al., 2004). The foundation of this neurodegeneration is an energy deficit induced by the blockade of trans-endothelial glucose transport (Osborne et al., 2015, Sapolsky, 1994). This metabolic restriction fosters a state of "energy starvation", against which GCs profoundly dysregulate neurotransmitter balance. They potentiate glutamate excitotoxicity by suppressing the function of astrocytic transporters (GLT-1), responsible for glutamate reuptake (Virgin et al., 1991, Popoli et al., 2011). An additional contributing mechanism is the stimulation of GC-induced neuroinflammation. Excess steroids are known to "prime" microglia for a hyperreactive state, resulting in an aberrantly high release of pro-inflammatory cytokines – including TNF-α, IL-1β, IL-6, and IL-18, as well as chemokines like CCL2 – even in response to minor stimuli (Frank et al., 2015). This cascade culminates in chronic oxidative stress, where mitochondrial dysfunction solidifies the cycle of pathological reactions.

The massive, uncontrolled influx of calcium ions into neurons, exacerbated by GC-mediated downregulation of calcium-binding protein expression, transforms mitochondria from primary energy-producing organelles into sources of reactive oxygen species (ROS). The subsequent development of aggressive oxidative stress and the activation of proapoptotic factors (such as caspase-3) lead to irreversible damage to the brain parenchyma and widespread neuronal death (Ohmoto et al., 2020). The ability of nervous tissue to withstand such a calcium "assault" and prevent the launch of the apoptosis program depends directly on the effectiveness of intracellular protective systems. Maintaining cellular Са^2+^-homeostasis relies not only on energy-dependent transport and compartmentalization mechanisms but also on a highly specific system for ion sequestration via Са^2+^-binding proteins (Park et al., 2019). This specific protein group functions as a primary defense mechanism, detecting even minor fluctuations in intracellular Ca^2+^ levels. Key CNS representatives include calbindin, calretinin, parvalbumin, and the glia-derived neurotrophic protein S100B, though others likely contribute to this regulatory network (Park et al., 2019, Hernández-Ortega et al., 2024). A unique position within this hierarchy is occupied by osteocalcin, an osteoprotein whose regulatory role in neural function is currently undergoing active reconsideration (Chen et al., 2026). Owing to their differential affinities for Са^2+^ (ranging from 10^-^⁹ to 10^-^⁶ М), these proteins serve a dual function: they not only provide immediate buffering of toxic excess calcium but also act as critical mediators within Са^2+^-dependent signaling pathways essential for neuronal survival under stress conditions (Park et al., 2019).

It is well established that long-term glucocorticoid administration, exacerbated by concurrent vitamin D_3_ deficiency, induces a pronounced destabilization of calcium homeostasis (Park et al., 2019, Kong et al., 2025). While the most documented consequence of this imbalance is glucocorticoid-induced osteoporosis, parallel degenerative processes simultaneously emerge within neural tissue (Reichardt et al., 2021, Shorey et al., 2023, Koning et al., 2024). Amidst the energetic crisis and excitotoxicity precipitated by corticosteroid loading, the capacity of neurons and glia to sequester excess calcium emerges as a critical determinant of cellular survival. Consequently, the role of Ca^2+^-sensory and Ca^2+^-binding proteins, alongside other neurospecific markers, in modulating the glial response remains an area of profound scientific interest.

The translation of molecular data into neuroprotective strategies requires the identification of specific biomarkers capable of quantifying brain damage at preclinical stages. While the clinical utility of markers such as neuron-specific enolase (NSE), myelin basic protein (MBP), and glial fibrillary acidic protein (GFAP) is well-established in acute neuropathologies (Comeau et al., 2023, Kocik et al., 2024, Makovec et al., 2025), their diagnostic relevance during protracted corticosteroid exposure remains insufficiently characterized. Under chronic hormonal loading, there is a pressing need to validate a more comprehensive panel of sensitive indicators, including neurofilament light chain (NF-L) and S100B, alongside MBP and GFAP. The translocation of these molecules into the systemic circulation serves as a high-fidelity, minimally invasive index of blood-brain barrier (BBB) permeability and neuro-glial disintegration.

It has been previously demonstrated that chronic oral prednisolone administration induces severe structural, neurophysiological, and behavioral disturbances in a rat model, establishing a clear link between vitamin D_3_ deficiency and functional CNS decline (Lisakovska et al., 2023). However, the precise underlying biochemical profiling of calcium-regulating systems remains to be fully characterized. In light of these premises, this study aimed to elucidate the role of calcium dyshomeostasis and structural-functional CNS damage in glucocorticoid-induced neurotoxicity. Central to this objective was the biochemical profiling of Ca^2+^-binding/sensory proteins (calbindin-D28K, S100B, osteocalcin) and neurospecific markers (NF-L, MBP, GFAP) in brain tissue and serum as indicators of injury and therapeutic response. The specific research tasks were: first, to evaluate the expression patterns of calbindin-D28K, S100B, and osteocalcin under chronic prednisolone exposure and determine the efficacy of vitamin D_3_ in restoring these levels; second, to assess reactive astrogliosis and demyelination by utilizing S100B, MBP, and GFAP as biomarkers for neuroinflammation and parenchymal injury; and third, to provide a pathogenetic rationale for the neuroprotective administration of vitamin D_3_ based on the restoration dynamics of these target proteins.

## 2 Materials and methods

### 2.1 Modeling of glucocorticoid-induced neurotoxicity and the vitamin D_3_ administration protocol

Experimental studies were conducted on female Wistar rats with an initial body weight of 100 ± 5 g. Female rats were selected due to their higher susceptibility to metabolic disturbances and secondary vitamin D_3_ deficiency during glucocorticoid treatment, reflecting the higher clinical prevalence of long-term glucocorticoid therapy among women. All animals were housed under standard vivarium conditions (temperature +22 ± 2°C, relative humidity 50–60%, 12/12-hour light/dark cycle, ad libitum access to water and standard granulated feed). Rodents were randomly assigned to the three experimental groups (n = 18 per group) using a weight-stratified randomization protocol to ensure homogenous baseline body weight across all groups: a control group, comprising intact animals receiving 0.1 mL of sunflower oil vehicle daily; a GC group, receiving synthetic glucocorticoid prednisolone (5 mg/kg body weight, aqueous suspension) and 0.1 mL of sunflower oil as a vehicle; and a correction group, receiving concurrent treatment with prednisolone and vitamin D_3_ (1000 IU/kg body weight, dissolved in 0.1 mL of sunflower oil). Prednisolone was preferred over more potent glucocorticoids (e.g., dexamethasone) to accurately model the gradual progression of neurotoxic and metabolic shifts characteristic of chronic oral maintenance therapy commonly used in clinical practice. All treatments were administered per os by oral gavage daily for 30 days.

Experimental procedures were conducted in strict accordance with national and international bioethical standards, including the “Guidance on the operation of the Animals (Scientific Procedures) Act 1986”, “EU Directive 2010/63/EU for the protection of animals used for scientific purposes”, and the national ethical guidelines (Law of Ukraine, 2006). The study protocol complied with the ARRIVE guidelines and was approved by the Institutional Bioethics Committee (Protocol No. 7 from September 20, 2023). Every effort was made to minimize animal suffering and to reduce the number of animals used to the statistically justified minimum, in compliance with the 3 R principles. To optimize tissue utilization and avoid unnecessary animal use, individual rats from each group (n = 18 total per group) were shared across different experimental assays. Specifically, at the end of the experiment, blood samples were collected from n = 10 randomly selected animals per group to perform serum biomarker analyses. Following euthanasia, the brains of these animals were harvested: n = 6 rats per group were designated for tissue ELISA and Western blot analysis (cerebral cortex and hippocampus), while another n = 6 rats per group were dedicated to immunohistochemistry and confocal microscopy. No sample pooling was performed; all assays represent individual biological replicates. Data collection, image acquisition during confocal microscopy, and quantitative analysis of immunoblots were performed by investigators blinded to the experimental group assignments to eliminate observer bias.

### 2.2 Collection and preparation of biological samples

At the end of the experimental period, blood was collected via cardiac puncture under light ether anesthesia (1.9% concentration). Samples were collected into tubes without anticoagulant and centrifuged at 3,000 rpm for 15 min at +4°C. The obtained serum was aliquoted and stored at −80°C.

Immediately after blood collection, animals were decapitated; the brain was removed, washed with chilled physiological saline, and the cerebral cortex and hippocampus were isolated on ice. For Enzyme-Linked Immunosorbent Assay (ELISA), brain tissues were homogenized in 10 volumes (w/v) of cold phosphate-buffered saline (PBS, pH 7.4) containing 0.1% Triton X-100 and a protease inhibitor cocktail (AEBSF, aprotinin, bestatin, E-64, leupeptin, and pepstatin A). The homogenates were incubated on ice for 30 min to ensure complete cell lysis, then centrifuged at 12,000 rpm for 20 min at +4°C. The resulting supernatants were collected as the soluble protein fraction.

For Western Blot analysis, tissues were lysed in cold RIPA buffer (50 mM Tris-HCl, pH 7.4, 150 mM NaCl, 1% NP-40, 0.5% sodium deoxycholate, 0.1% SDS) supplemented with a protease and phosphatase inhibitor cocktail. To achieve maximum protein solubilization, the samples were sonicated (3 cycles of 10 s at 40 kHz) and incubated on ice for 20 min. The lysates were then centrifuged at 14,000 rpm for 20 min at +4°C to remove insoluble debris. The concentration of total protein in all samples was determined using the Lowry method. The final supernatants (lysates) were aliquoted and stored at −80°C until further analysis.

### 2.3 Enzyme-linked immunosorbent assay

The concentrations of 25(OH)D_3_, NF-L, S100B, MBP, and GFAP in serum and brain tissue homogenates were quantified using commercial ELISA kits in accordance with the manufacturers' protocols. The following kits were utilized: Rat Vitamin D total (DE1971; Demeditec Diagnostics GmbH, Germany), Rat NF-L (NBP2–81185, Novus Biologicals, USA), Rat S100B (MBS2505625, MyBioSource, USA), Rat MBP (NBP2–81167, Novus Biologicals, USA), and Rat GFAP (NS830, Millipore, USA).

The validity of these assays for brain tissue matrices was confirmed via linearity-of-dilution tests. High-concentration cerebral samples were serially diluted (1:2–1:16), demonstrating strong linear recovery (R² > 0.98) within the dynamic range of each assay. Quantitative analysis of target protein levels was performed using the GainData® digital platform (arigo's ELISA Calculator). All tissue-specific markers were normalized to the total protein content of each sample, with final levels expressed as units of mass per milligram of total protein (pg/mg or ng/mg protein).

### 2.4 Western blot analysis

The expression levels of osteocalcin, calbindin-D28K, tumor necrosis factor-alpha (TNF-α), and interleukin-1 beta (IL-1β) were analyzed in brain tissue lysates. Samples containing 50 µg of total protein were denatured in Laemmli sample buffer at +95°C for 5 min. Proteins were separated by 10–15% SDS-PAGE (depending on their molecular weight) and electroblotted onto nitrocellulose membranes (Bio-Rad, USA) using a wet transfer tank. To prevent non-specific binding, membranes were blocked with 5% non-fat dry milk in PBS containing 0.05% Tween-20 (PBST) for 1 h at room temperature.

Membranes were then incubated overnight at +4°C with the following primary antibodies: mouse anti-osteocalcin (1:500, sc-365797, Santa Cruz Biotechnology, USA), mouse anti-calbindin-D28K (1:500, sc-365360, Santa Cruz Biotechnology, USA), mouse anti-IL-1β (1:1000, sc-52012, Santa Cruz Biotechnology, USA), and rabbit anti-TNF-α (1:1000, ARC3012, Invitrogen, USA). After washing three times with PBST, the membranes were incubated for 1 h with horseradish peroxidase (HRP)-conjugated secondary antibodies: anti-mouse IgG (1:2000, A9917, Sigma, USA) or anti-rabbit IgG (1:1000, #1706515, Bio-Rad, USA). Protein bands were visualized using an enhanced chemiluminescence (ECL) substrate (Thermo Scientific, USA). The optical density of the bands was quantified using GelPro Analyzer 3.2 software.

To avoid protein detachment and signaling artifacts associated with chemical stripping, normalization was achieved via parallel gel electrophoresis. Briefly, identical aliquots of protein samples were loaded onto duplicate SDS-PAGE gels and resolved simultaneously under identical experimental conditions. One membrane was incubated with primary antibodies against target proteins, while the parallel duplicate membrane was probed for 1 h at room temperature with HRP-conjugated β-actin antibody (1:10000, A3854, Sigma-Aldrich, USA) to control for loading consistency. β-actin served as a stable internal loading control, demonstrating uniform expression across all groups. Full uncropped blot images are available in the Supplementary Material.

### 2.5 Immunohistochemistry and confocal microscopy

Fixed brain sections (cortex and hippocampus) underwent standard deparaffinization and rehydration. Heat-induced epitope retrieval was performed in 10 mM citrate buffer (pH 6.0) at +95°C for 20 min. To prevent non-specific binding, sections were blocked with 5% normal goat serum in PBS containing 0.3% Triton X-100 for 1 h. Sections were then incubated overnight at +4°C with primary antibodies: rabbit anti-NG2 (1:250, PA5–100235, Invitrogen, USA) or mouse anti-GFAP (1:250, MA5–12023, Invitrogen, USA). After washing, sections were incubated for 1 h at room temperature with appropriate fluorescent-labelled secondary antibodies: goat anti-Rabbit IgG (H+L) Secondary Antibody, conjugated with DyLight 488 (1:250, #35552, Invitrogen, USA) or goat anti-mouse IgG H&L, conjugated with Alexa Fluor 568 (1:1000, ab175473, Abcam, UK). Fluorescence was visualized and imaged using a Carl Zeiss LSM 510 Meta confocal microscope. The number of positive cells was quantified in the cerebral cortex (layers II–V) and the hippocampal CA1 region within a standardized area of 200,000 µm². For each animal, 5 non-consecutive coronal sections were analyzed. Within each section, 3 random fields of view were examined in the target regions. Sampling was performed systematically, and quantitative analysis was executed by an investigator blinded to the treatment groups.

### 2.6 Statistical analysis

Statistical analysis was performed using the OriginPro 8.5 software package (OriginLab Corporation, Northampton, MA, USA). The normality of data distribution was confirmed using the Shapiro-Wilk test. Results are presented as the arithmetic mean ± standard deviation (SD). To determine the statistical significance of differences between the three experimental groups, one-way analysis of variance (one-way ANOVA) was applied, followed by Tukey’s post hoc test for multiple comparisons. The threshold for statistical significance was set at p < 0.05.

## 3 Results

### 3.1 Systemic and local levels of 25(OH)D_3_

Prolonged 30-day administration of prednisolone resulted in a significant decrease in the levels of the primary circulating vitamin D_3_ metabolite, 25(OH)D_3_, in both blood serum and brain tissue (Fig. 1). In the GC-treated group, the serum 25(OH)D_3_ concentration was 1.9-fold lower (63.29 ± 12.35 nmol/L, p < 0.001) than in the control group (121.01 ± 17.77 nmol/L; Fig. 1A). A similar trend was observed in the brain (Fig. 1B), where 25(OH)D_3_ levels declined 2.3-fold (8.38 ± 1.33 ng/g vs. 19.05 ± 2.38 ng/g in the control, p < 0.001). Concurrent administration of vitamin D_3_ partially restored these parameters to 106.93 ± 16.79 nmol/L in serum and 14.15 ± 2.30 ng/g in brain tissue, representing a significant increase compared to the prednisolone-only group (p < 0.001 for both). These data demonstrate that chronic prednisolone exposure induces a substantial systemic and local vitamin D_3_ deficiency.

**Fig. 1:**
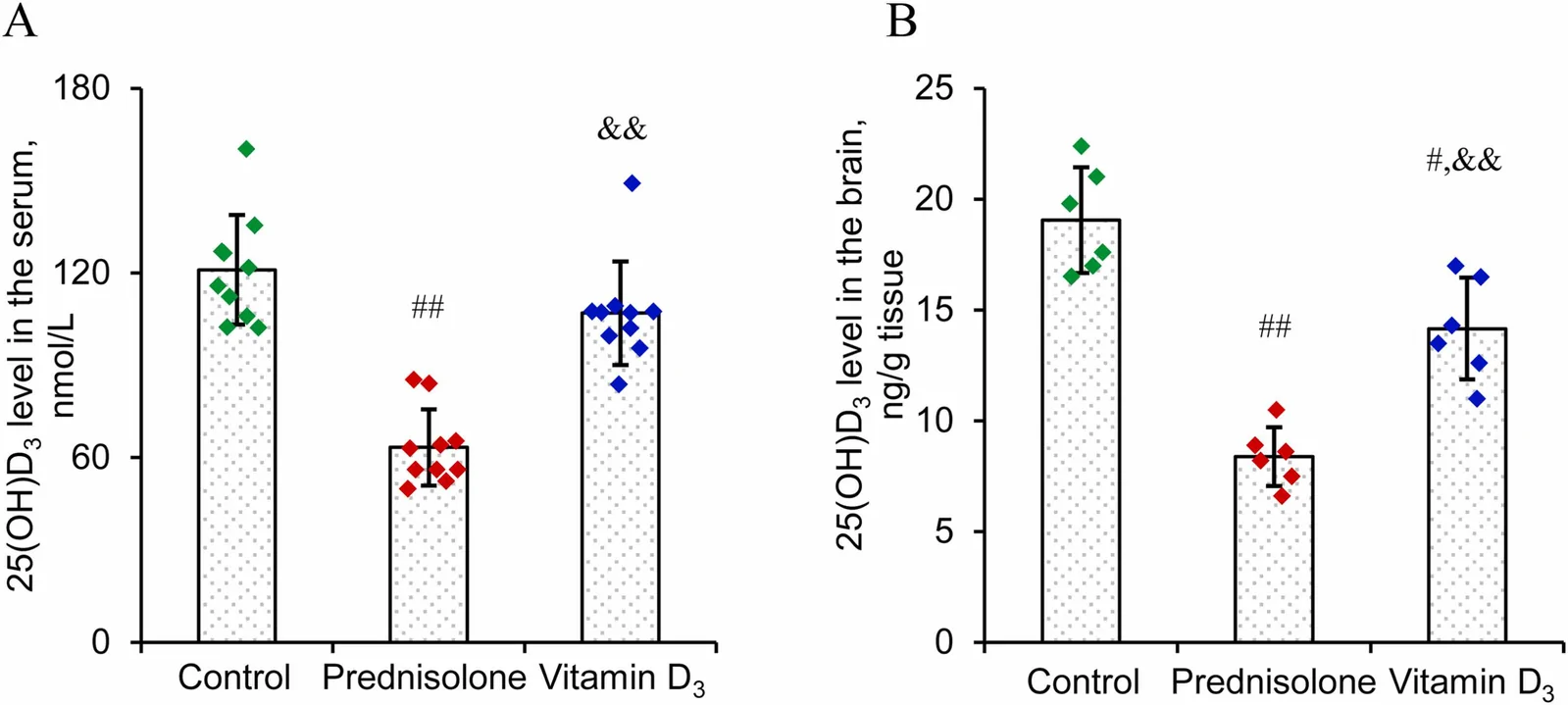
Effects of prednisolone and vitamin D_3_ on 25-hydroxyvitamin D_3_ (25(OH)D_3_) levels. Concentrations of 25(OH)D_3_ in blood serum (A, n = 10) and brain homogenates (B, n = 6) were measured by ELISA. All data are presented as mean ± SD. Statistical significance is denoted as: ^#^ p < 0.05, ^##^ p < 0.001 vs^.^ control; ^&&^ p < 0.001 vs. GC group^.^

### 3.2 Changes in Ca^2+^-binding protein profiles in the rat brain

The observed vitamin D_3_ deficiency was associated with altered levels of calcium-binding proteins. Analysis of these markers revealed multidirectional changes under glucocorticoid-induced neurotoxicity (Fig. 2).

**Fig. 2:**
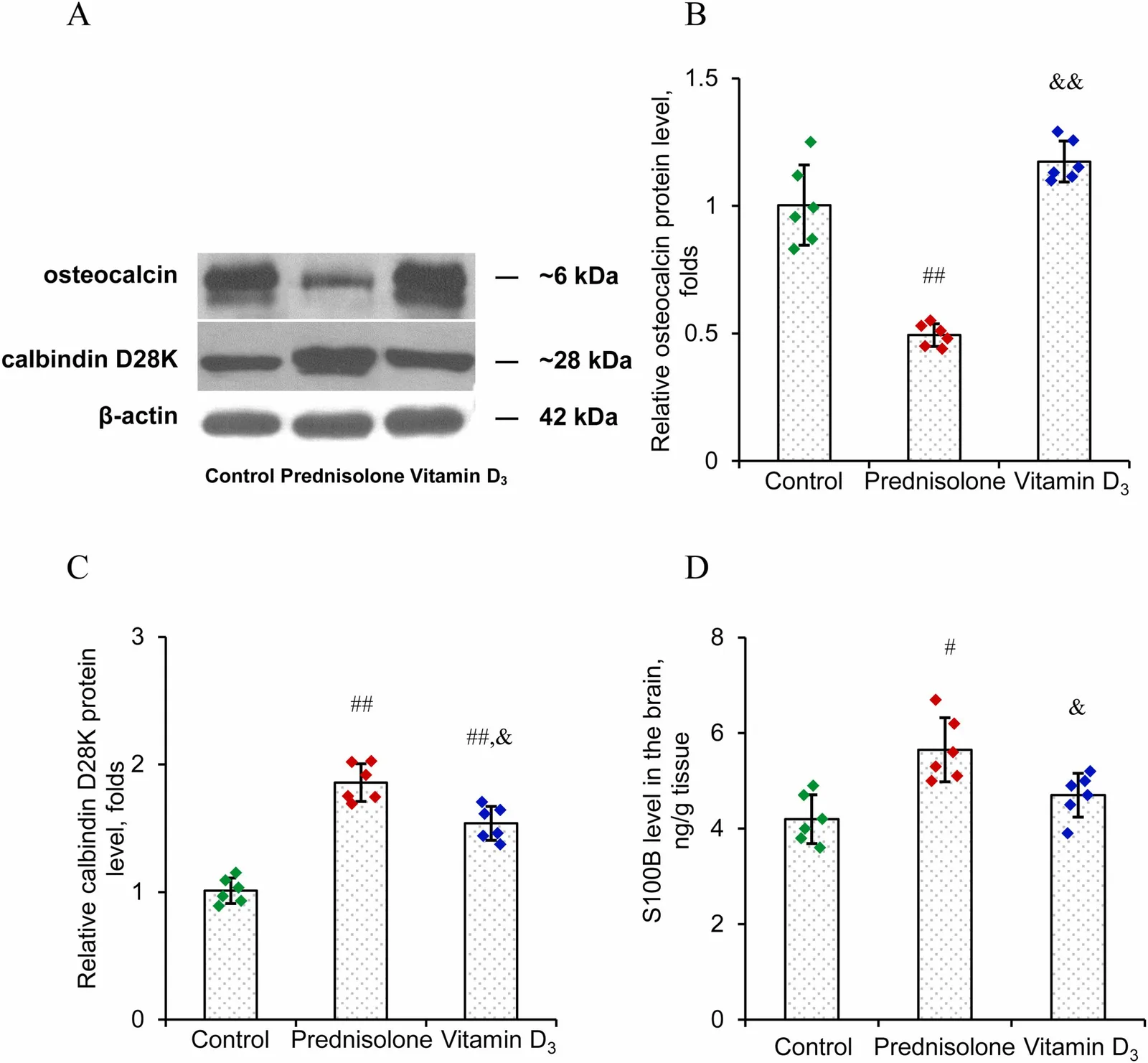
Protein levels of Ca^2+^-binding and Ca^2+^-sensing markers in the rat brain. (A) Representative immunoblots and quantitative analysis of (B) osteocalcin and (C) calbindin-D28K protein levels (n = 6). Data were normalized to β-actin. (D) Content of glial cell-specific protein S100B in brain homogenates determined by ELISA (n = 6). All data are presented as mean ± SD. Statistical significance is denoted as: ^#^ p < 0.05, ^##^ p < 0.001 vs^.^ control; ^&^ p < 0.05, ^&&^ p < 0.001 vs^.^ GC group.

Specifically, a twofold decrease in osteocalcin protein content (Fig. 2A,B) was observed in the brain following prolonged prednisolone administration (p < 0.001 vs. control). Vitamin D_3_ supplementation normalized osteocalcin levels, with no significant difference remaining compared to the control group (p = 0.053). Conversely, calbindin-D28K levels (Fig. 2A,C) exhibited a marked 1.85-fold increase in the brain (p < 0.001 vs. control). Vitamin D_3_ treatment only partially mitigated this effect, resulting in an 18% decrease compared to the GC group (p = 0.002); however, levels remained 1.53-fold higher than the baseline control (p < 0.001). The level of the glial cell-specific protein S100B (Fig. 2D) was 33% higher in the brains of the GC group (5.65 ± 0.67 ng/g vs. 4.20 ± 0.51 ng/g in the control, p = 0.001). Vitamin D_3_ administration reduced S100B content by 17% compared to the GC group (4.70 ± 0.46 ng/g, p = 0.025), effectively reaching control values (p = 0.29).

These results demonstrate that prednisolone exposure disrupts the balance of calcium-binding proteins. While vitamin D_3_ effectively restores osteocalcin and S100B levels, it exerts only a limited effect on the glucocorticoid-induced elevation of calbindin-D28K.

### 3.3 Impact of prednisolone and vitamin D_3_ on myelination and glial cell populations

Analysis of myelination and astrogliosis markers revealed distinct structural alterations in nerve tissue. Under neurotoxic conditions, the level of myelin basic protein (Fig. 3A) showed a moderate 16% decrease (5.50 ± 0.49 µg/g vs. 6.55 ± 0.69 µg/g in the control, p = 0.035). Therapeutic administration of vitamin D_3_ did not significantly affect MBP content (5.93 ± 0.75 µg/g, p = 0.501 vs. the GC group). The population of NG2^+^ oligodendrocyte precursors in the hippocampal CA1 region (Fig. 3B,C) decreased 1.6-fold following glucocorticoid exposure, from 42 ± 4 cells/200,000 µm² in the control to 27 ± 3 cells/200,000 µm² (p < 0.001). Vitamin D_3_ treatment facilitated a partial recovery of this cell count (33 ± 4 cells/200,000 µm², p = 0.042 vs. GC group), although the number of NG2^+^ cells remained 21.4% lower than in intact animals (p = 0.003). No significant changes in NG2^+^ cell counts were observed in the cerebral cortex across any groups (Fig. 3B, D; p > 0.05).

**Fig. 3:**
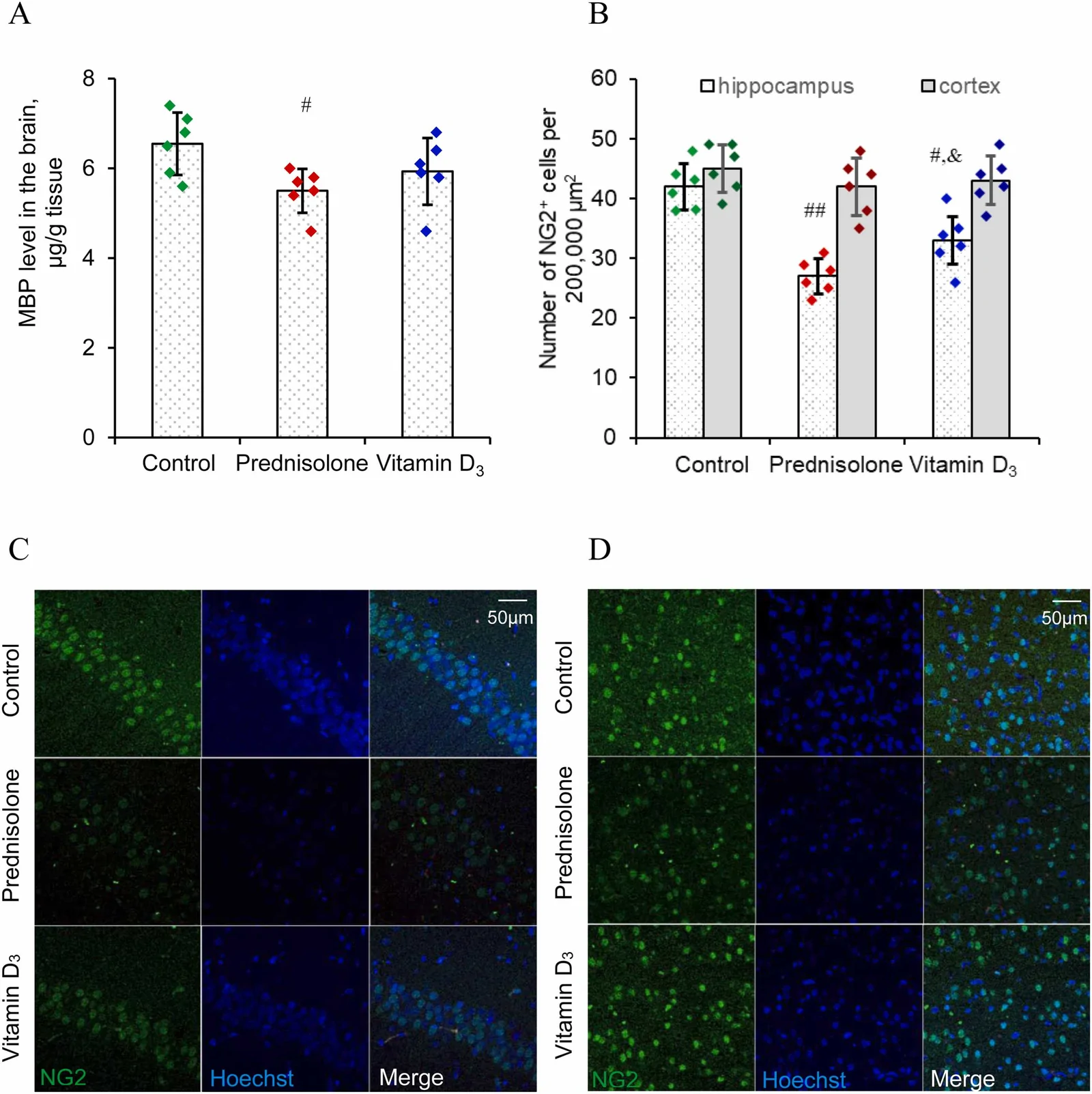
Myelination marker and oligodendrocyte precursor populations. (A) Myelin basic protein (MBP) concentration in brain homogenates (n = 6; ELISA). (B) Quantification of NG2^+^ oligodendrocyte precursors in the hippocampus and cerebral cortex (n = 6). Representative fluorescence microscopy images of NG2-positive cells in the hippocampal CA1 region (C) and cerebral cortex (D) (magnification ×400, scale bar = 50 μm). Data are mean ± SD. ^#^ p < 0.05, ^##^ p < 0.001 vs^.^ control; ^&^ p < 0.05 vs. GC group.

The astrocyte population (GFAP^+^ cells) significantly increased in the cerebral cortex (32 ± 4 cells/200,000 µm²) and hippocampus (47 ± 3 cells/200,000 µm²), representing 1.60- and 1.81-fold increases, respectively, compared with intact animals (20 ± 2.3 cells/200,000 µm² and 26 ± 3 cells/200,000 µm²; p < 0.001; Fig. 4A–C). Vitamin D_3_ administration significantly reduced the number of reactive astrocytes (25 ± 2 cells/200,000 µm² in the cortex, p = 0.002; 36 ± 5 cells/200,000 µm² in the hippocampus, p < 0.001) compared with the GC group. Interestingly, total GFAP levels in brain lysates remained unchanged across all groups (Fig. 4D), suggesting localized cellular redistribution or reactive morphological changes rather than an alteration in total tissue protein concentration.

**Fig. 4:**
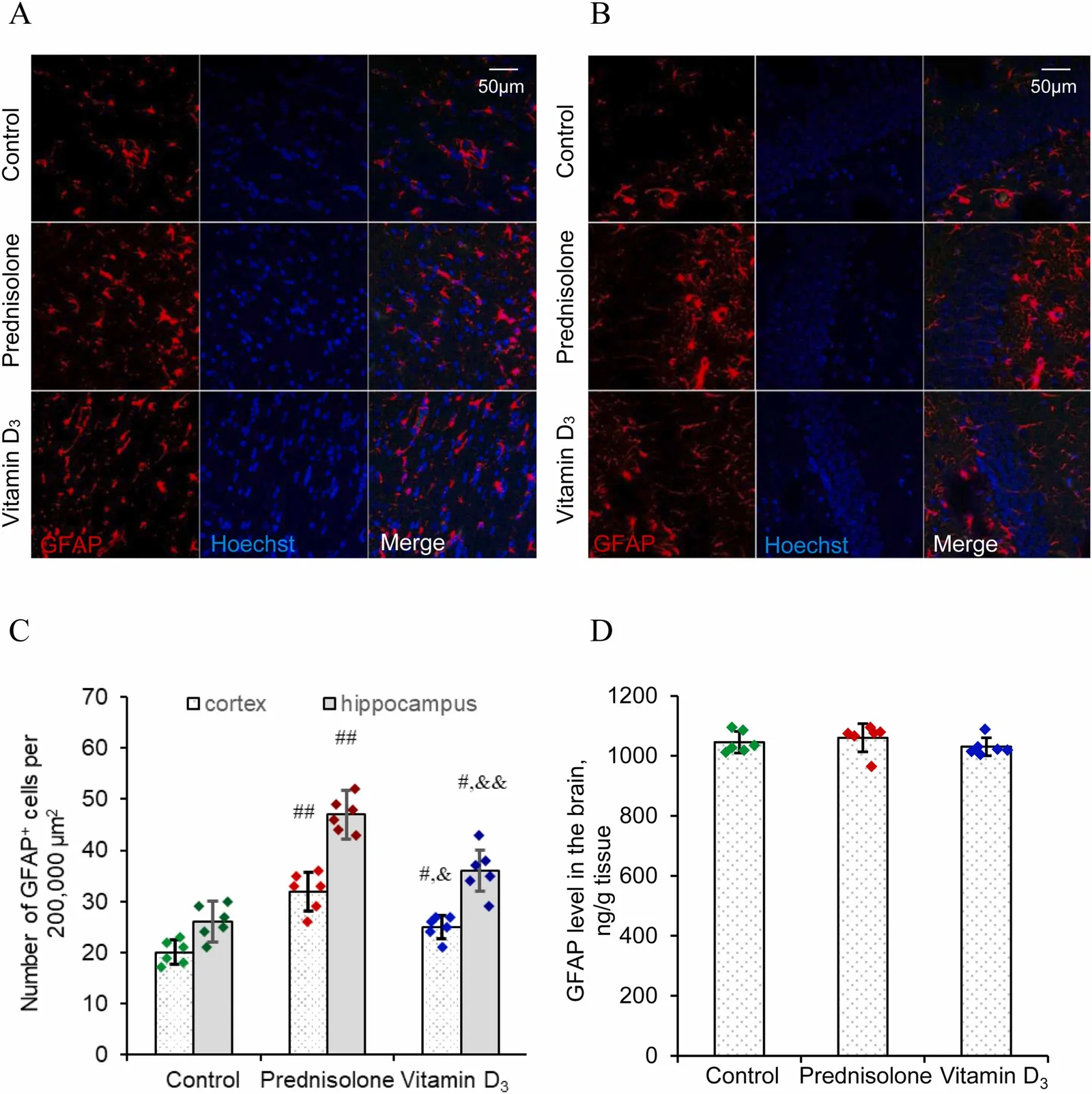
Glial cell response to prednisolone and vitamin D_3_ treatment. Representative fluorescence images of GFAP-positive astrocytes in the cerebral cortex (A) and hippocampal C1 region (B) (magnification ×400, scale bar = 50 μm). (C) Quantification of the astrocyte population based on GFAP marker (n = 6). (D) GFAP concentration in brain homogenates (n = 6; ELISA). Data are mean ± SD. ^#^ p < 0.05, ^##^ p < 0.001 vs^.^ control; ^&^ p < 0.05, ^&&^ p < 0.001 vs^.^ GC group.

These findings demonstrate that glucocorticoids alter myelin integrity, trigger astrogliosis, and deplete the hippocampal pool of oligodendrocyte precursors. While vitamin D_3_ selectively modulates astrocyte reactivity and facilitates the partial restoration of NG2^+^ cells, it exhibits a limited capacity to reverse the glucocorticoid-induced reduction of MBP.

### 3.4 Proinflammatory cytokine profiles (TNF-α and IL-1β) in brain tissue

Beyond disruptions in calcium-binding proteins and glial alterations, the development of neuroinflammation is a key element of GC-induced neurotoxicity. Prolonged prednisolone administration led to a significant increase in proinflammatory cytokine levels across brain structures. Specifically, TNF-α content (Fig. 5A,B) rose in both the cerebral cortex (1.62-fold, p < 0.001) and hippocampus (1.65-fold, p < 0.001). Even more substantial elevations were observed for IL-1β (Fig. 5A,C), which increased 2.66-fold in the cortex and 2.40-fold in the hippocampus (p < 0.001 vs. control for both). These findings reflect the intensive activation of the neuroinflammatory cascade under steroid-induced stress.

**Fig. 5:**
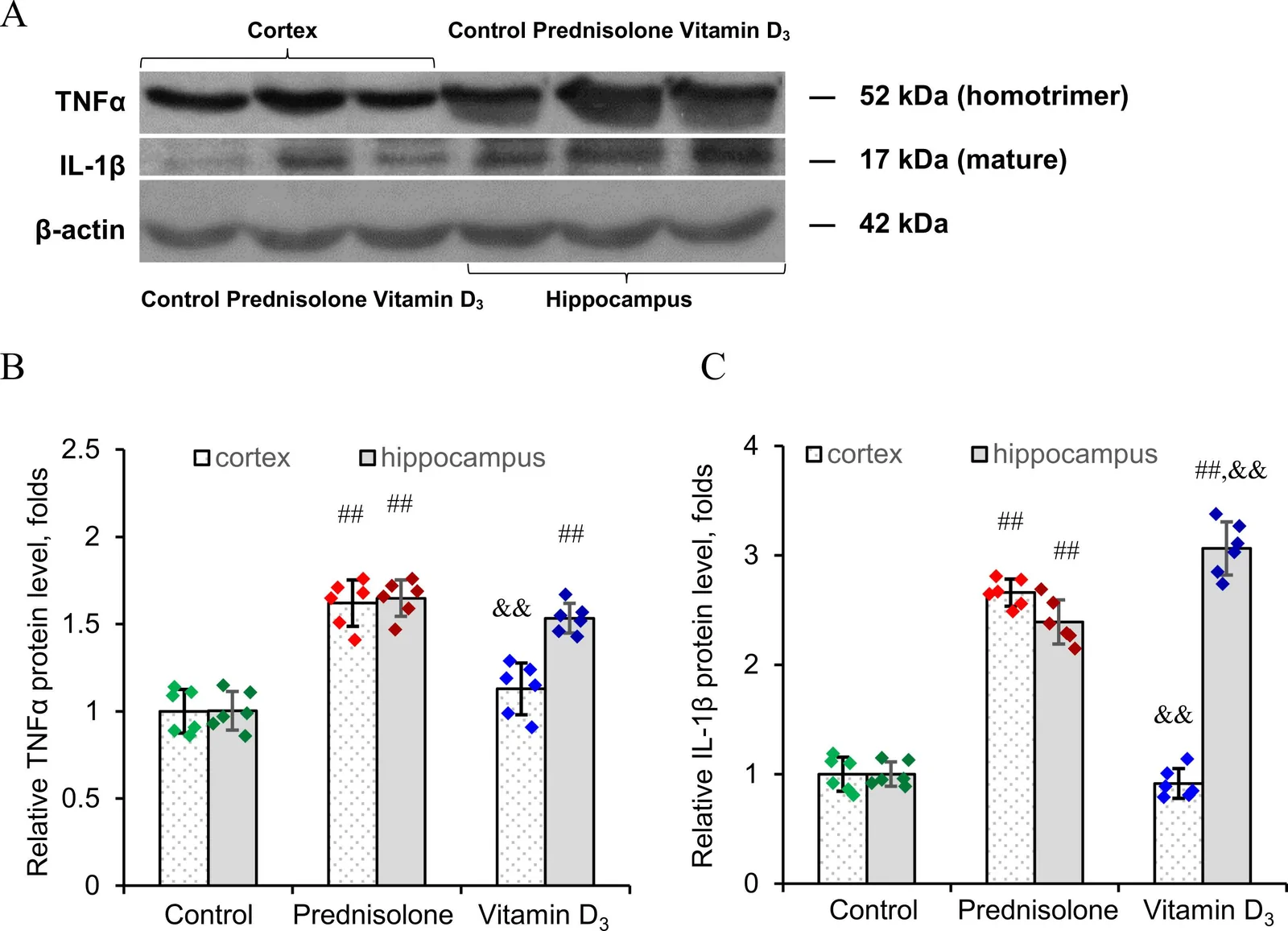
Proinflammatory cytokine levels across brain regions. (A) Representative immunoblots and quantitative analysis of (B) TNF-α and (C) IL-1β protein levels in the cerebral cortex and hippocampus (n = 6). Relative protein content was normalized to β-actin. Data are mean ± SD. ^##^ p < 0.001 vs. control; ^&&^ p < 0.001 vs^.^ GC group.

Vitamin D_3_ administration demonstrated varying efficacy in modulating these markers depending on the brain region. In the cerebral cortex, TNF-α levels were restored to control values, and IL-1β content normalized (p = 0.263 and p = 0.553, respectively, vs. intact animals). However, in the hippocampus, the reduction of TNF-α compared with the GC group was not statistically significant (p = 0.151). Notably, hippocampal IL-1β levels remained elevated 3.06-fold compared to the control (p < 0.001) and showed a further 1.27-fold increase compared to the prednisolone-only group (p < 0.001).

In summary, vitamin D_3_ exerts a pronounced anti-inflammatory effect in the cerebral cortex by neutralizing the glucocorticoid-induced surge of TNF-α and IL-1β, while demonstrating limited effectiveness in the hippocampus. These results indicate that brain regions exhibit differential sensitivity to both the toxic effects of glucocorticoids and the therapeutic intervention of cholecalciferol.

### 3.5 Serum levels of neurospecific damage biomarkers

To identify potential diagnostic criteria for CNS damage during chronic glucocorticoid exposure, levels of neuron-specific proteins in biological fluids were evaluated. While neurofilament light chain levels in brain extracts (Fig. 6A) remained unchanged across groups (p > 0.05), a significant increase in NF-L was detected in the blood serum (610.32 ± 101.73 pg/mL vs. 458.62 ± 45.16 pg/mL in the control, p = 0.011) under glucocorticoid-induced stress (Fig. 6B). This elevation likely reflects the severity of axonal neurodegeneration. Cholecalciferol exerted a corrective effect on circulating NF-L levels (483.25 ± 39.27 pg/mL), decreasing them by 20.8% compared with the prednisolone group (p = 0.033).

**Fig. 6:**
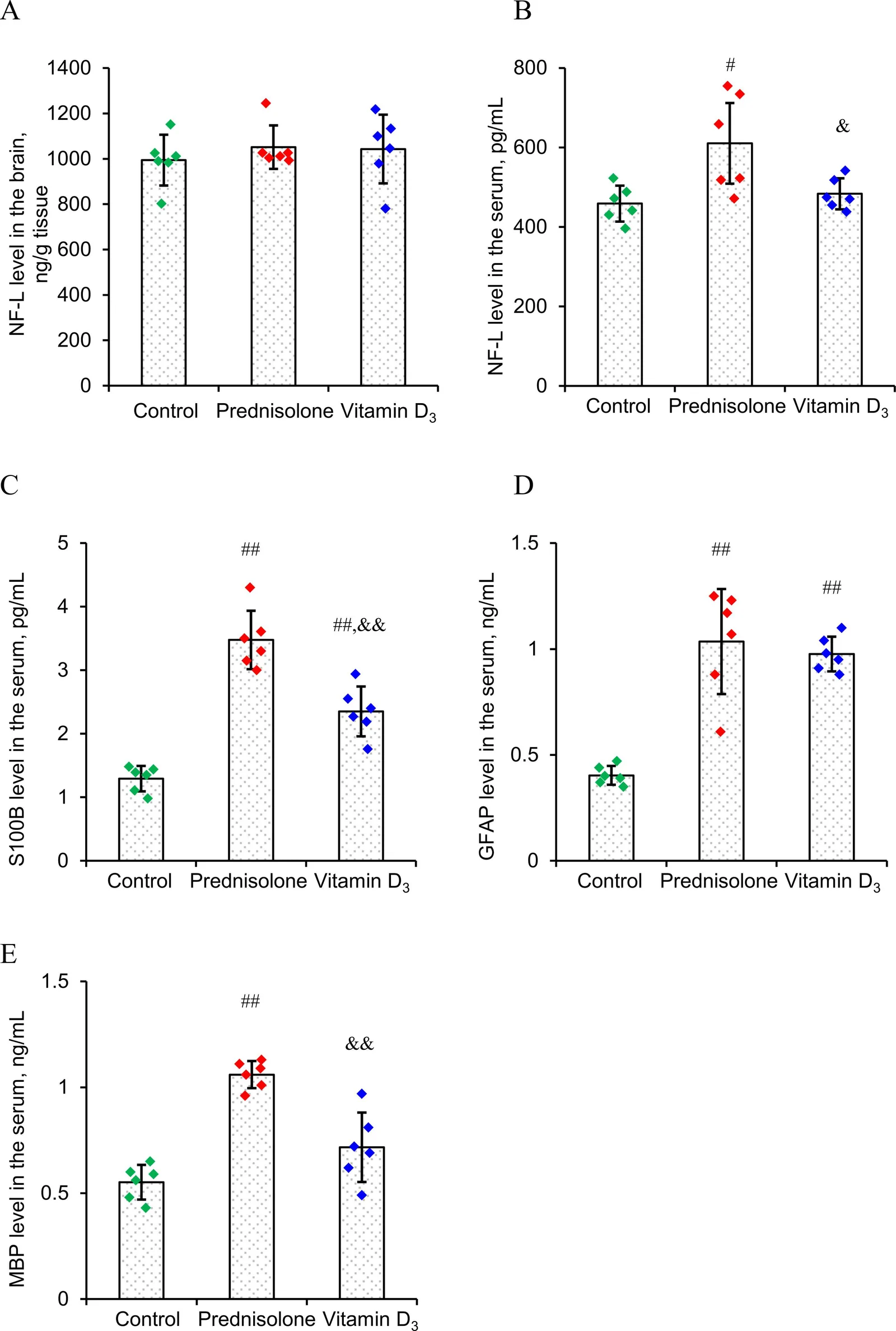
Analysis of neurospecific biomarkers in brain tissue and serum. Concentration of neurofilament light chain (NF-L) in brain extracts (A) and serum levels of (B) NF-L, (C) S100B, (D) GFAP, and (E) MBP measured by ELISA (n = 6). All data are presented as mean ± SD. Statistical significance is denoted as: ^#^ p < 0.05, ^##^ p < 0.001 vs^.^ control; ^&^ p < 0.05, ^&&^ p < 0.001 vs^.^ GC group.

Regarding other markers, serum S100B levels (Fig. 6C) increased 2.7-fold (3.48 ± 0.46 pg/mL vs. 1.29 ± 0.20 pg/mL in the control, p < 0.001) but decreased by 32.5% following vitamin D_3_ administration to 2.35 ± 0.39 pg/mL (p < 0.001 vs. the prednisolone group). Serum GFAP levels (Fig. 6D) increased 2.6-fold (1.04 ± 0.25 ng/mL vs. 0.40 ± 0.04 ng/mL in healthy animals, p < 0.001), indicating alterations consistent with blood-brain barrier dysfunction; notably, cholecalciferol did not significantly reduce serum levels of this protein (0.98 ± 0.08 ng/mL, p = 0.79 vs. GC group). Finally, a nearly twofold increase in serum MBP (Fig. 6E) was detected after prednisolone administration (1.06 ± 0.06 ng/mL vs. 0.55 ± 0.08 ng/mL in the control, p < 0.001). Vitamin D_3_ therapy significantly attenuated MBP levels to 0.72 ± 0.16 ng/mL (a 32% reduction, p < 0.001 vs. GC group).

These data suggest that serum levels of NF-L, S100B, GFAP, and MBP may reflect CNS alterations and potential blood-brain barrier dysfunction, serving as indirect indicators to track the dynamics of vitamin D_3_ therapy.

## 4 Discussion

The present research comprehensively elucidates the mechanisms underlying the neurotoxic effects of long-term glucocorticoid therapy and provides a detailed analysis of the neuroprotective potential of vitamin D_3_ in mitigating such destructive processes. It was established that prolonged administration of prednisolone induces a critical deficiency of vitamin D_3_, evident both systemically (in the blood serum) and locally within the brain tissue. Our data align with existing literature, which indicates a significantly elevated risk of vitamin D_3_ deficiency in patients undergoing steroid therapy (Davidson et al., 2012). In agreement with current understanding of the metabolic antagonism between GCs and cholecalciferol, glucocorticoids can act as potent inducers of active vitamin D_3_ catabolism via VDR-mediated expression of 24-hydroxylase (Zayny et al., 2019). Consequently, this enzymatic shift leads to a sharp decrease in the bioavailability of 25(OH)D_3_, the primary circulating metabolite measured in our study, which inevitably limits the synthesis of active 1α,25(OH)_2_D_3_. The resulting depletion provokes a negative calcium balance and impairs intestinal calcium absorption. Such a metabolic shift triggers a cascade of pathomorphological changes, consistent with our previous histological observations of profound structural brain damage and neurophysiological disturbances in the present model (Lisakovska et al., 2023).

In this context, vitamin D_3_ deficiency might act as a pivotal trigger for systemic CNS destabilization; the initial disruption of calcium homeostasis, mediated by the dysregulation of calcium-binding proteins, could potentially form the basis for secondary neuroinflammation and progressive demyelination. A salient diagnostic and pathogenetic indicator of the observed processes is the aberrant expression of proteins such as calbindin-D28K and S100B, which exhibit a distinctly divergent pattern. The recorded multidirectional changes may reflect a tissue-specific response to glucocorticoid-induced stress, where the reduction in osteocalcin likely indicates systemic metabolic suppression, while the upregulation of calbindin-D28K appears to serve as a compensatory mechanism to buffer a toxic excess of cytosolic Ca^2+^. Specifically, a significant suppression of osteocalcin levels is observed, reflecting systemic osteoblast suppression and bone matrix degradation within the classical context of glucocorticoid-induced osteoporosis. Conversely, a compensatory upregulation of calbindin-D28K content is recorded in the nervous tissue. This phenomenon can be interpreted as an acute adaptive response of neurons designed to buffer excess cytosolic Ca^2+^ during excitotoxic stress. Nevertheless, such an increase should be interpreted cautiously, as it may also reflect an underlying cellular stress response or an altered neuronal phenotype induced by chronic steroid exposure. Without adequate vitamin D_3_ – the primary regulator of calcium-binding protein synthesis – the adaptive buffering might become exhausted. Consequently, cells could lose their ability to sequester toxic calcium concentrations, which potentially activate calcium-dependent proteases and may initiate irreversible apoptotic pathways.

Of particular scientific value is the analysis of recovery dynamics within calcium-regulating protein systems following vitamin D_3_ administration. The observed complete normalization of osteocalcin levels indicates the high metabolic lability of the mentioned marker and its critical dependence on exogenous vitamin D_3_ supplementation. Conversely, the only partial correction of calbindin-D28K levels suggests that GC-induced neurotoxicity causes a deeper and more stable regulatory shift. The incomplete restoration of the latter could potentially result from prolonged damage to the neuronal genetic apparatus or the depletion of antioxidant potential, necessitating extended therapy or the recruitment of additional neuroprotective agents. Consequently, such a lack of full recovery within the calcium signaling system warrants a detailed evaluation of structural and functional brain damage through the prism of the glial response. The detection of neurospecific proteins S100B and GFAP serves as a fundamental biochemical indicator of reactive changes within the CNS, as their elevated levels directly correlate with glial cell activation and the initiation of the neuroinflammatory cascade (Zayny et al., 2019, Makovec et al., 2025). Interestingly, while the number of GFAP-positive cells increased significantly in the cortex and hippocampus, the total GFAP protein concentration in brain lysates remained unchanged. This apparent discrepancy likely reflects a reactive morphological transformation (astrocytic hypertrophy, elongation, and redistribution of processes) typical of mild-to-moderate astrogliosis, rather than a massive de novo synthesis of the protein across the entire tissue matrix.

Within the pathophysiological framework, the calcium-binding protein S100B functions as a damage-associated molecular pattern (DAMP); upon reaching high extracellular concentrations, it interacts with receptors for advanced glycation end-products (RAGE) and Toll-like receptors (TLRs) (Kato and Svensson, 2015). Ligand-receptor binding subsequently triggers pro-inflammatory signaling pathways, potentially leading to chronic neuroinflammation and secondary neuronal degradation. The underlying mechanism of such destruction is closely linked to disrupted calcium homeostasis in glial cells, particularly astrocytes, where an abnormal increase in cytosolic Ca^2+^ activates calcineurin, subsequently stimulating the production and release of pro-inflammatory cytokines (Leal et al., 2004). Furthermore, a key component of this neuroinflammatory cascade is likely the activation of the NLRP3 inflammasome, which orchestrates the cleavage of procaspase-1 to active caspase-1, leading to the maturation and secretion of potent pro-inflammatory cytokines, notably IL-1β and IL-18 (Xu et al., 2025). Excess GCs may "prime" microglia toward hyperreactivity, potentially triggering an abnormally high release of pro-inflammatory mediators even under minor stimuli, thus creating conditions for chronic oxidative stress (Feng et al., 2019).

In contrast to the aforementioned destructive processes, vitamin D_3_ exhibits pronounced neuroprotective and immunomodulatory properties, functioning as a potent regulator of the CNS microenvironment. Recent studies confirm that vitamin D_3_ can mitigate the pathological activation of reactive astrocytes and microglia by suppressing pro-inflammatory mediators and contributing to the restoration of nervous tissue functionality (Sangha et al., 2023). Such a neuroprotective action is likely realized through the capacity of cholecalciferol to modulate immune cell activity and stabilize intracellular calcium turnover. Importantly, it has been previously demonstrated that chronic prednisolone exposure triggers a significant activation of the NF-κB signaling pathway in the rat brain, which was effectively suppressed by concurrent vitamin D_3_ administration (Lisakovska et al., 2023). Based on these established data, we hypothesize that the downstream anti-inflammatory effects observed in the current study, such as the attenuation of IL-1b and TNF-a maturation, might be directly driven by this cholecalciferol-mediated NF-κB inhibition. While the components of the NLRP3 inflammasome, including ASC (apoptosis-associated speck-like protein containing a CARD) and caspase-1, or TXNIP regulation were not directly measured in the present work, accumulating evidence indicates that vitamin D_3_ can potentially modulate these immune pathways as well (Abdelrahman et al., 2023, Camargo et al., 2020, Hoseinrad et al., 2021, Wang et al., 2024). Consequently, further mechanistic studies focusing on gene silencing or Western blotting for cleaved caspase-1 remain essential to definitively validate the full interplay between NF- κB and the NLRP3 cascade in our specific model.

Crucially, cholecalciferol treatment is hypothesized to potentially alleviate the impairment of hippocampal glucocorticoid receptors induced by chronic steroid exposure. According to Camargo et al. (2020), this tentative effect might be associated with the reduction of TXNIP and other inflammasome-related proteins, which could facilitate the restoration of receptor homeostasis and mitigate depressive-like behaviors. The efficacy of this intervention is partially supported by the marked reduction in S100B and GFAP levels observed in our study, signaling the attenuation of reactive astrogliosis. Unexpectedly, concurrent vitamin D_3_ supplementation led to a further increase in hippocampal IL-1b levels. While the exact mechanism remains to be elucidated, this could suggest a regional hyper-reactivity of hippocampal microglia or a transient pro-inflammatory paradox potentially linked to the specific dosage of cholecalciferol used. Alternatively, the 30-day window might capture a transitional phase of tissue remodeling where transient immune activation and microglial clearance of cellular debris precede the resolution of inflammation. This distinct regional disparity highlights that the hippocampus may possess distinct VDR/GR sensitivity profiles and a different temporal profile of immune balance compared to the cerebral cortex. Alternatively, achieving a robust therapeutic threshold in this stress-sensitive architecture may warrant optimized pharmacological concentrations or a more sustained supplementation period.

Parallel to neuroinflammation, a critical aspect of CNS degradation is the disruption of white matter integrity. The observed decrease in MBP levels and the reduction in the NG2^+^ oligodendrocyte precursor population represent direct evidence of myelin sheath destruction and suppressed regenerative potential. In such a context, vitamin D_3_ demonstrated selective effectiveness: while promoting the partial restoration of the NG2^+^ pool, it did not significantly impact MBP levels. This discrepancy suggests that the recovery of the cellular precursor reserve precedes terminal differentiation and active myelin synthesis. The limited effect on MBP contrasts with studies where high-dose vitamin D_3_ led to pronounced remyelination (Al-Otaibi et al., 2023, Nystad et al., 2018), a process mediated by the stimulation of oligodendrocyte maturation. Thus, the absence of an immediate MBP increase despite positive NG2^+^ dynamics likely stems from a temporal lag between cellular regeneration and biochemical recovery, suggesting that complete remyelination may necessitate a more extended duration of therapy.

The findings of the present study confirm the hypothesis that vitamin D_3_ acts as a potent neurosteroid and auto-/paracrine regulator, whose role extends far beyond classical mineral metabolism. Its ability to modulate the levels of calcium-binding proteins, stabilize the glial microenvironment, and exert anti-inflammatory effects positions it as an indispensable component in strategies aimed at mitigating the side effects of prolonged glucocorticoid therapy. Accordingly, a systemic reflection of the recorded intracerebral disorders is the transformation of the biochemical profile of the blood serum, which is of fundamental importance for assessing pathological dynamics. The increased extra-cerebral concentrations of NF-L, S100B, and GFAP indicate substantial alterations consistent with dysfunction of the brain's barrier functions (Tefr Faridova et al., 2023). Upon entering the systemic bloodstream, the detected markers function not only as indicators of large-scale glial activation and axonal degeneration but also as indirect indices of structural integrity alterations of the blood-brain barrier. It should be noted, however, that the assessment of BBB integrity in the present study relies on the circulating macromolecules without direct permeability assays such as Evans Blue extravasation or tight junction protein profiling. Therefore, these serum shifts should be interpreted as surrogate markers consistent with BBB dysfunction rather than direct evidence of physical barrier breakdown.

According to existing data, matrix metalloproteinases (specifically MMP-9 and MMP-2) play a pivotal role in BBB degradation by cleaving type IV collagen – the primary component of the capillary basement membrane – and tight junction proteins, such as claudin-5 and occludin (Blecharz et al., 2010, Shen et al., 2024). Although glucocorticoids may stabilize the barrier during acute phases, chronic exposure or neurotoxic concentrations often provoke a pro-inflammatory shift, leading to the pathological activation of MMPs. The latter facilitates the extravasation of neurospecific macromolecules into the general circulation and enhances immune cell infiltration into the brain parenchyma (Blecharz et al., 2010). The established correlation between the suppression of serum indicators under the influence of vitamin D_3_ and the regression of tissue degradation markers suggests the potential involvement of cholecalciferol in restoring BBB permeability. Recent studies indicate that vitamin D_3_ may act as a potent inhibitor of MMP-9 expression while stimulating the synthesis of tight junction proteins, which effectively "seals" the barrier and restricts the efflux of neurodegeneration biomarkers (Akinrinde et al., 2025, Niapour et al., 2024).

The described mechanism holds high translational significance and is fully consistent with our findings of a pathogenetic link between the dysfunction of the vitamin D_3_ auto-/paracrine system and structural CNS disorders. Prospective research should now focus on establishing dose-dependent effects to define the optimal therapeutic window for promoting terminal oligodendrocyte differentiation and myelin resynthesis. Additionally, further investigation into the specific modulation of the MMP-9/TIMP balance and tight junction protein assembly within this experimental model is warranted to fully elucidate the processes governing BBB repair. Finally, long-term clinical studies are essential to validate the serum profile of neurospecific proteins as a reliable predictive panel across various age groups, potentially establishing these markers as non-invasive tools for personalized neuroprotective support.

Several limitations of the present research must be acknowledged. First, the absence of a vitamin D_3_-alone control group restricts our ability to isolate the baseline independent neurobiological effects of cholecalciferol from its direct counter-regulatory actions against glucocorticoids. Second, while the analysis identified key changes in cytokine profiles and glial markers, the downstream molecular interactions were inferred based on protein expression rather than assessed via gene silencing or knockout models. Third, although functional behavioral and cognitive evaluations were outside the scope of the present biochemical profiling, it has been previously established that this exact protocol of chronic prednisolone exposure leads to pronounced locomotor deficits, anxiety, and cognitive decline in rats (Lisakovska et al., 2023). The current findings expand upon that functional phenotype by identifying the molecular and surrogate barrier mechanisms driving those deficits. Fourth, this study was conducted exclusively on female Wistar rats due to their higher clinical susceptibility to metabolic steroid complications. Consequently, potential sex-specific confounding factors, including the estrous cycle, circulating sex hormones, and sex-dependent variations in vitamin D metabolism, were not controlled for and highlight the need for future investigation in mixed-sex cohorts.

In summary, the results obtained confirm the hypothesis that vitamin D_3_ acts as a potent neurosteroid and auto-/paracrine regulator, whose role extends far beyond classical mineral metabolism (Cui and Eyles, 2022). Its ability to influence the expression of calcium-buffering proteins, stabilize the glial microenvironment, and restore the neurovascular interface state positions it as an indispensable component in strategies aimed at mitigating the side effects of prolonged glucocorticoid therapy. The integrated analysis of serum biomarkers allows for the real-time objectification of CNS integrity, which convincingly justifies the therapeutic expediency of monitoring and correcting vitamin D_3_ status in patients receiving long-term steroid treatment.

## 5 Conclusions

Prolonged glucocorticoid exposure induces a complex array of neurodegenerative changes characterized by a significant reduction in local and systemic vitamin D_3_ levels, the onset of neuroinflammation, disturbances in intracellular calcium regulation, and myelin degradation. Elevated serum levels of CNS-associated proteins, specifically S100B, GFAP, NF-L, and MBP, may serve as potential biomarkers consistent with blood-brain barrier dysfunction and the subsequent systemic leakage of neurospecific macromolecules. Furthermore, glucocorticoid excess triggers a compensatory increase in neuronal calbindin-D28K within brain tissue, reflecting a significant adaptive response to disrupted intracellular calcium buffering.

Vitamin D_3_ therapy demonstrates robust neuroprotective potential by effectively normalizing serum levels of osteocalcin, S100B, and MBP, while reducing reactive astrogliosis and partially restoring the oligodendrocyte precursor (NG2^+^) population. The potent ability of cholecalciferol to stabilize calcium metabolism and maintain the regenerative pool of oligodendrocytes highlights its critical role in preserving the structural and functional integrity of the CNS under conditions of steroid-induced neurotoxicity. These findings provide preclinical evidence justifying the monitoring of vitamin D_3_ status in patients undergoing long-term glucocorticoid therapy.

## Institutional review board statement

Animal care during experimental procedures was carried out in strict accordance with national and international bioethical standards, including the “Guidance on the operation of the Animals (Scientific Procedures) Act 1986”, “EU Directive 2010/63/EU for the protection of animals used for scientific purposes”, and the national ethical guidelines (Law of Ukraine, 2006). The study protocol complied with the ARRIVE guidelines and was approved by the Institutional Bioethics Committee (Protocol No. 7 from September 20, 2023).

## Ethics declaration

This study was conducted in accordance with the ARRIVE (Animal Research: Reporting of In Vivo Experiments) guidelines. This study was approved by the The Committee of Biosafety and Bioethics of the Palladin Institute of Biochemistry of NAS of Ukraine. (Approval No. 7 from September 20, 2023)

## Funding

This work was supported by the National Research Foundation of Ukraine (project No. 2021.01/0417).

## CRediT authorship contribution statement

**Ihor Shymanskyi:** Writing – original draft, Validation, Project administration, Funding acquisition, Data curation, Conceptualization. **Olha Lisakovska:** Writing – review & editing, Visualization, Software, Methodology, Investigation, Formal analysis. **Anna Khomenko:** Writing – review & editing, Validation, Methodology, Investigation. **Vasyl Bilous:** Writing – review & editing, Investigation. **Mykola Veliky:** Writing – review & editing, Supervision, Resources, Conceptualization.

## Conflicts of Interest

The authors declare that they have no known competing financial interests or personal relationships that could have appeared to influence the work reported in this paper.

## Data Availability

The data that support the findings of this study are available from the corresponding author upon reasonable request.
