# Supplementary material for: Neuroprotective potential of vitamin D3 against glucocorticoid-induced neurotoxicity: Focus on calcium-binding proteins and neuroinflammation in a rat model
Source: IBRO Neurosci Rep. 2026 Sep 26;21:922–33. doi: 10.1016/j.ibneur.2026.09.016 (PMC13656948; doi:10.1016/j.ibneur.2026.09.016)

Supplementary Figure

The whole uncropped images of the original western blots

1) Figure 2A – Osteocalcin

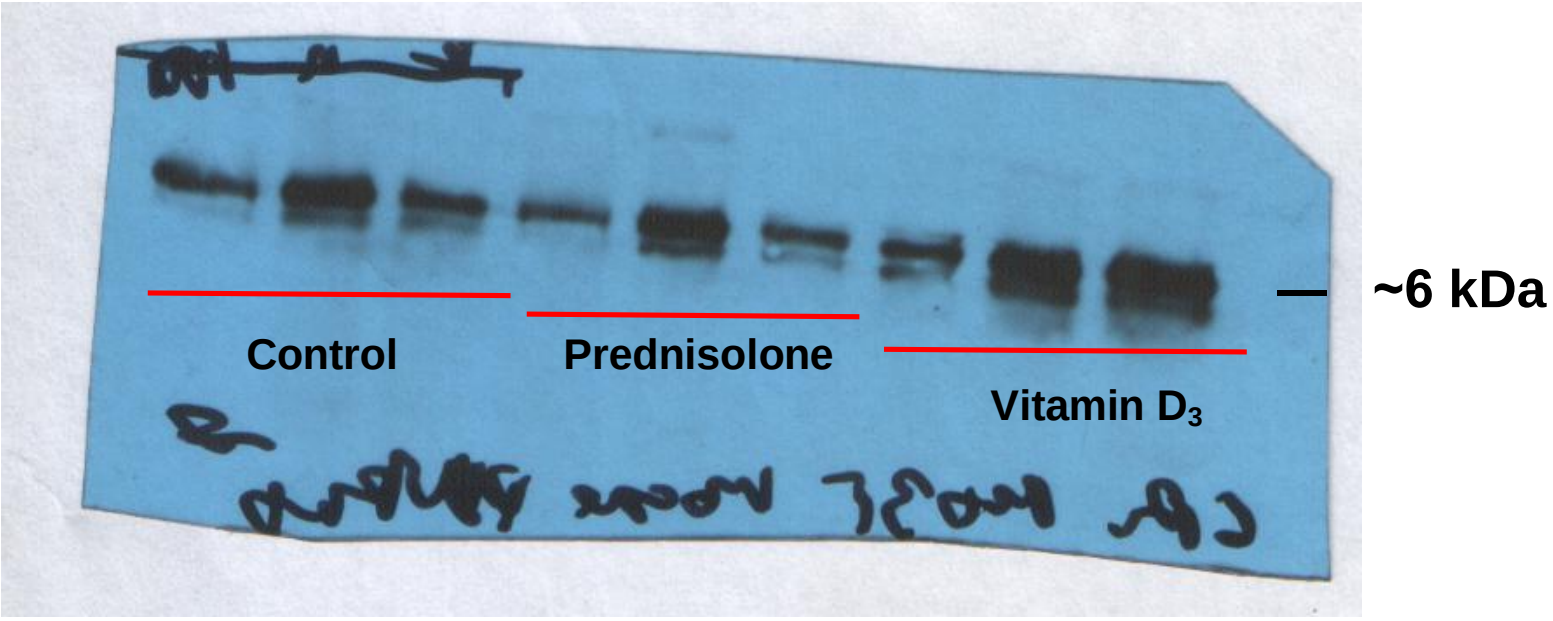

Figure 2A – Calbindin D28K

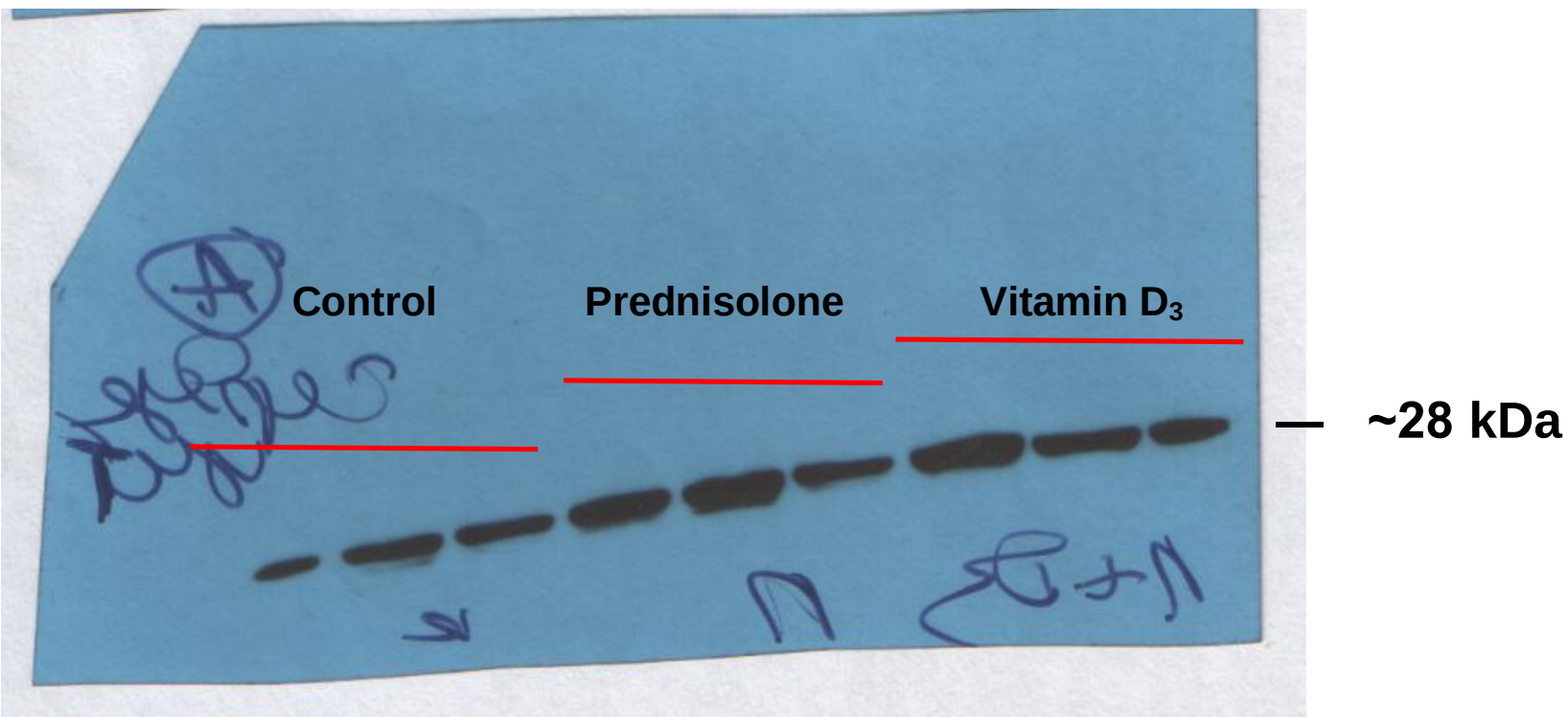

Figure 2A – beta-actin

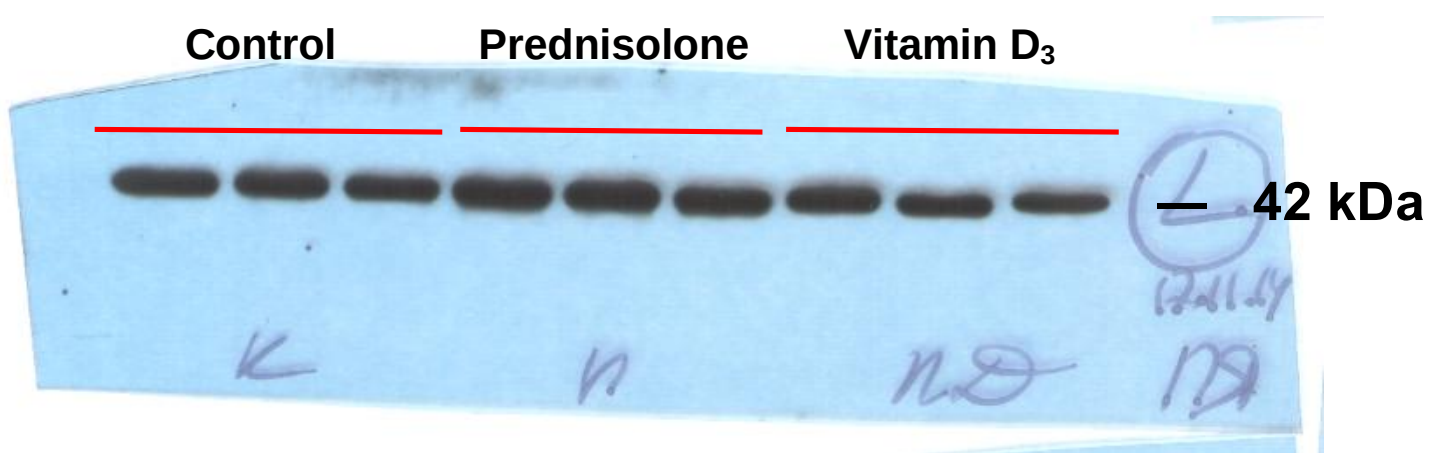

2) Figure 5A – TNFα

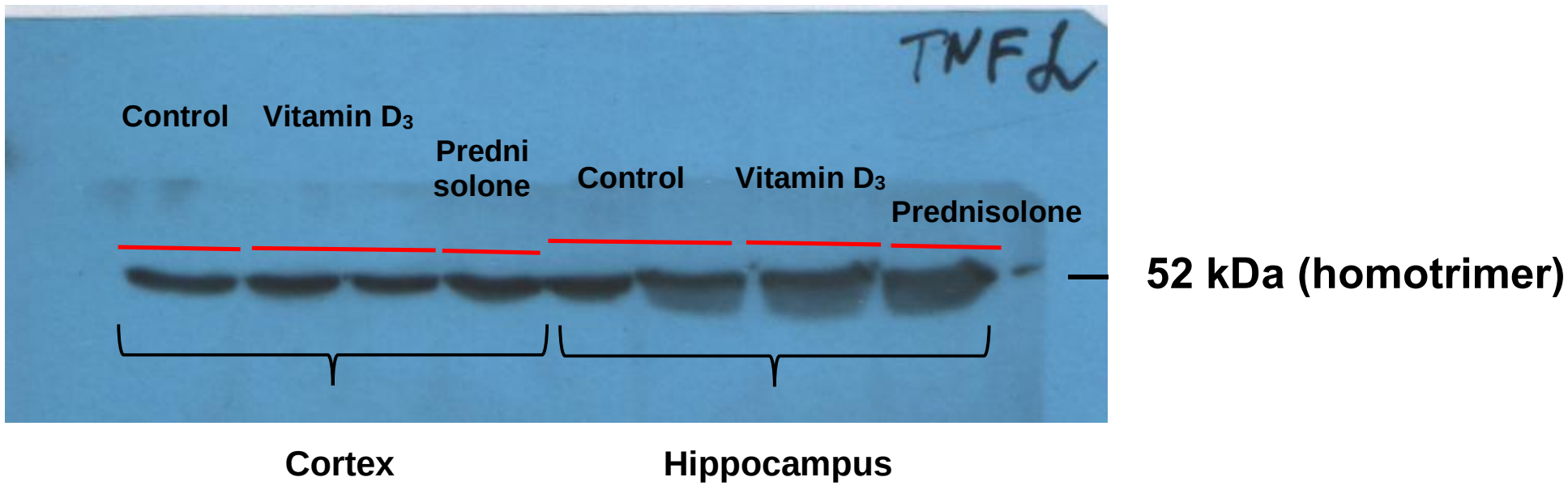

Figure 5A – IL-1β

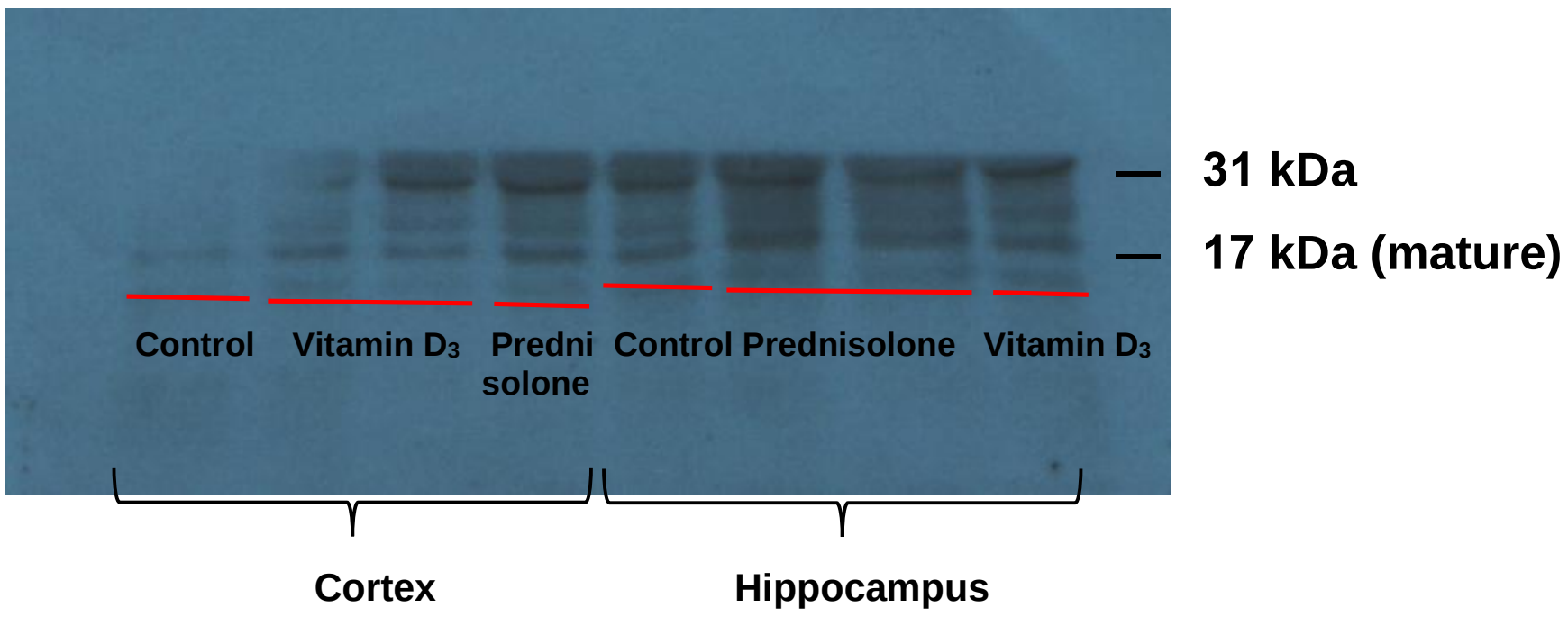

Figure 5A – beta-actin

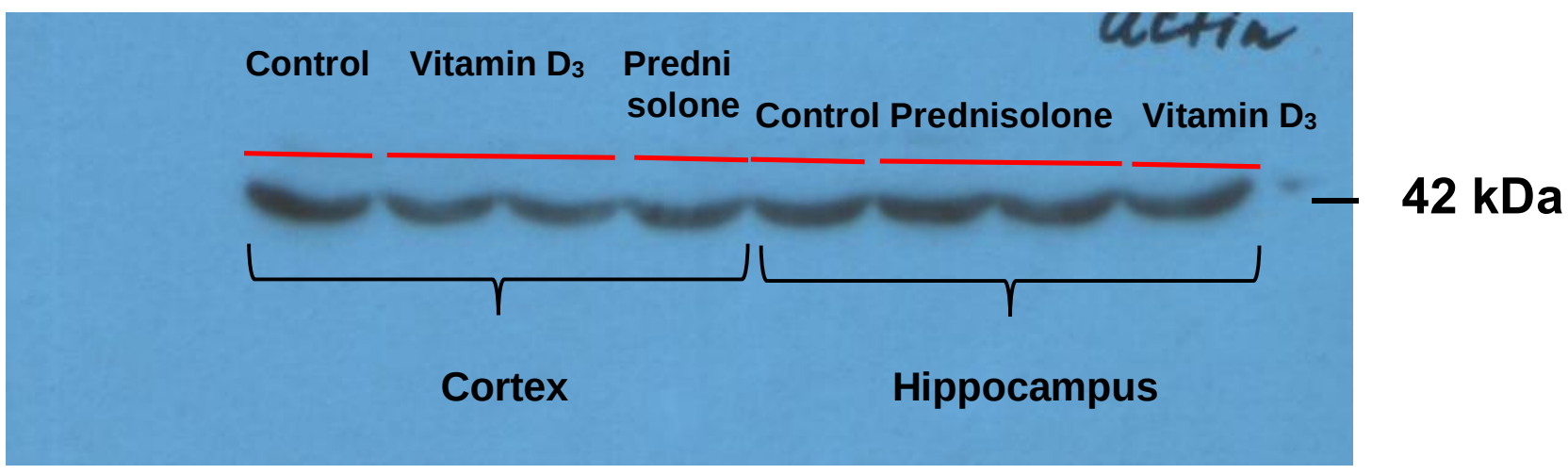

Supplement: Supplementary file 1 [file mmc1.pdf]
